# Retrospective accuracy analysis of MRI based lesion size measurement in neuroblastic tumors: which sequence should we choose?

**DOI:** 10.1186/s12880-020-00503-1

**Published:** 2020-09-10

**Authors:** Sebastian Gassenmaier, Ilias Tsiflikas, Simon Maennlin, Cristian Urla, Steven W. Warmann, Juergen F. Schaefer

**Affiliations:** 1 Department of Diagnostic and Interventional Radiology, University Hospital Tuebingen, Hoppe-Seyler-Straße 3, 72076 Tuebingen, Germany; 2 Department of Pediatric Surgery and Pediatric Urology, University Children’s Hospital Tuebingen, Tuebingen, Germany

**Keywords:** MRI, Neuroblastoma, Histopathology, Diagnostic accuracy, Fibrosis

## Abstract

**Background:**

MR imaging of neuroblastic tumors is widely used for assessing the effect of chemotherapy on tumor size. However, there are some concerns that MRI might falsely estimate lesion diameters due to calcification and fibrosis. Therefore, the aim of our study was to compare neuroblastic tumor size based on MRI measurements to histopathology measurements of the resected specimens as standard of reference.

**Methods:**

Inclusion criteria were diagnosis of a neuroblastic tumor, MR imaging within 100 days to surgery and gross total resection without fragmentation of the tumor between 2008 and 2019. Lesion diameters were measured by two radiologists according to RECIST 1.1 in axial plane in T2w turbo spin echo (TSE), diffusion-weighted imaging (DWI), and in T1w pre- and postcontrast sequences. Furthermore, the largest lesion size in three-dimensions was noted. The largest diameter of histopathology measurements of each specimen was used for comparison with MRI.

**Results:**

Thirty-seven patients (mean age: 5 ± 4 years) with 38 lesions (neuroblastoma: *n* = 17; ganglioneuroblastoma: *n* = 11; ganglioneuroma: *n* = 10) were included in this retrospective study. There was excellent intra-class correlation coefficient between both readers for all sequences (> 0.9) Tumor dimensions of reader 1 based on axial MRI measurements were significantly smaller with the following median differences (cm): T1w precontrast − 1.4 (interquartile range (IQR): 1.8), T1w postcontrast − 1.0 (IQR: 1.9), T2w TSE: -1.0 (IQR: 1.6), and DWI -1.3 (IQR: 2.2) (*p* < 0.001 for all sequences). However, the evaluation revealed no significant differences between the three-dimensional measurements and histopathology measurements of the resected specimens regardless of the applied MRI sequence.

**Conclusions:**

Axial MRI based lesion size measurements are significantly smaller than histopathological measurements. However, there was no significant difference between three-dimensional measurements and histopathology measurements of the resected specimens. T2w TSE and T1w postcontrast images provided the lowest deviation and might consequently be preferred for measurements.

## Background

Neuroblastoma (NB) is the most common extracranial malignant solid tumor in infants [1, 2]. Radiological imaging plays a pivotal role for diagnosis, risk stratification, and assessment of response to chemotherapy [3]. Thus, imaging is not only used for differentiation between the malignant NB, the less malignant ganglioneuroblastoma (GNB), and the benign ganglioneuroma (GN) but also for risk stratification according to the International Neuroblastoma Risk Group (INRG) classification system and staging system (INRGSS) [3–9]. Imaging modalities used for assessment involve ultrasound (US), computed tomography (CT), magnetic resonance imaging (MRI), and scintigraphy [3, 10]. The evaluation of response to chemotherapy prior to surgical resection is of paramount importance [11]. According to the INRGSS, the tumor size should be determined via a three-dimensional (3D) measurement of the tumor with CT or MRI [5]. However, this contradicts the very common Response Evaluation Criteria In Solid Tumors (RECIST) which state that only the largest diameter should be taken into account [12, 13]. Brodeur et al. proposed a 3D measurement for staging and response assessment due to the irregular tumor shape [14]. However, Bagatell et al. could show that there is no clear advantage in 3D measurements in comparison to one dimension regarding response assessment [15]. The determination of the exact tumor size is not only valuable for response evaluation but also for surgical planning and postoperative analysis. For residual tumor assessment the discrepancy between the resected specimens and preoperative measurements is of utmost importance. Additionally, in follow-up examinations of residual tumor the correctness of lesion size measurements is indispensable for the process of local disease progression.

Both CT and MRI can be used for staging of neuroblastic tumors [3]. However, due to the technical advances with rapid imaging and the radiation free process many factors are in favor of MRI for routine staging [11]. However, it is still unclear which MRI sequence is suited best for lesion size measurements. Furthermore, it was recently shown that MRI may underestimate the exact tumor size in abdominal neuroblastoma [16].

Therefore, the aim of this study was to compare the accuracy of MRI based lesion size measurement in neuroblastic tumors in different sequences with histopathology as standard of reference.

## Methods

### Study design

A retrospective, monocentric analysis of patients suffering from pediatric neuroblastic tumors and who were operated between 2008 and 2019 was carried out. The patients were identified using the institution’s radiology information system. The patients were included in the study if they fulfilled the following criteria: at least one available MRI study with a maximum of 100 days prior to surgery, gross total resection without fragmentation of the tumor mass, and complete histopathological work-up including histology as well as measurement of the tumor size. In case of several available MRI examinations, the most current one was used. Since many patients were referred from other countries to our clinic, in some cases a delay between the most current MRI examination and surgery was unavoidable. Due to the necessity of anesthesia and the risks involved, MRI examinations were only repeated in our department if absolutely necessary for surgery. The institutional review board approval for this study was obtained.

### Histopathology

All resected specimens were processed by the institutional pathology department. In case of uncertainness of the exact diagnosis a reference center was consulted for further clarification. The measurement data were extracted from the histopathology report. The largest diameter only was used as reference standard for comparison with MRI measurements.

### MRI based lesion size measurement

Because many patients were referred from external centers only for resection to our clinic, no uniform imaging protocol was available. Two measurement approaches were followed: Firstly, the diameter of the lesion was measured in axial plane according to RECIST 1.1 as available in T1 weighted (T1w) pre- and postcontrast imaging, T2 weighted (T2w) turbo spin echo (TSE) imaging, and diffusion-weighted imaging (DWI) [13]. In a second step, the largest diameter in three dimensions of the tumor was also noted to determine the overall largest size. Measurements were performed independently by one radiologist with 4 years of experience in post-processing procedures as well as by one radiologist with one year of experience in pediatric radiology with a standard software (syngo.via, Siemens Healthineers, Erlangen; Germany). The radiologists were blinded to the histopathological report. The largest diameter of each sequence in axial plane as well as in three dimensions was used for comparison with the histopathology results of the resected specimens. Mean and absolute differences between MRI and measurements of the resected specimens were calculated. After a gap of at least 2 weeks the measurements were carried out a second time by the same radiologists to determine intra-reader variability.

### Statistical analysis

Statistical analysis was performed using Jmp14 (SAS Institute, Cary, North Carolina; USA) and MedCalc Version 18.1 (MedCalc Software bvba, Ostend; Belgium). Due to the low sample size only non-parametric tests as the Wilcoxon signed rank test and Friedman test were used for paired data. The Pearson correlation coefficient was applied to test the relationship between MRI and histopathology measurements of the resected specimens. Intra-class correlation coefficient (ICC) was used to assess inter- and intra-rater variability. Bland-Altman plots were calculated to analyse the difference between MRI and histopathology measurements of the resected specimens.

The significance level alpha was set at 0.05.

## Results

### Patients’ characteristics

Thirty-seven patients fulfilled the inclusion criteria. The histopathological diagnoses were NB in 16 cases (including one patient with bilateral NB), GNB in 11 cases, and GN in 10 cases. There were 38 measurable lesions. Nineteen patients underwent surgical resection without preoperative chemotherapy while the remaining 18 patients received neoadjuvant chemotherapy prior to the operation.

Mean patient age at operation was 5 ± 4 years (range 0–16). Median time period between surgical resection and MRI was 18 days (interquartile range (IQR): 27; range 1–98). Further characteristics are displayed in Table 1.

**Table 1:** Patients’ characteristics

Characteristics	Values
Patients	n = 37 (*n* = 38 lesions)
Mean age ± std.	5 ± 4 years
Range	0–16 years
Time period MRI – Surgery	Median 18 days; interquartile range 27 days
*Diagnosis*	
Neuroblastoma	*n* = 16 (one bilateral resulting in 17 lesions)
Ganglioneuroblastoma	n = 11
Ganglioneuroma	n = 10
*Tumor localization*	
Cervical	n = 3
Thoracic	n = 3
Thoraco-abdominal	n = 3
Abdominal	*n* = 27 (one bilateral)
Pelvic	n = 1
*Treatment approach*	
Neoadjuvant therapy	*n* = 18 patients with 19 lesions (NB: *n* = 11; GNB: *n* = 8)
Directly referred to surgery	*n* = 19 patients (NB: n = 6; GNB: n = 3; GN: n = 10)

### Intra- and inter-reader variability

**Fig. 1:**
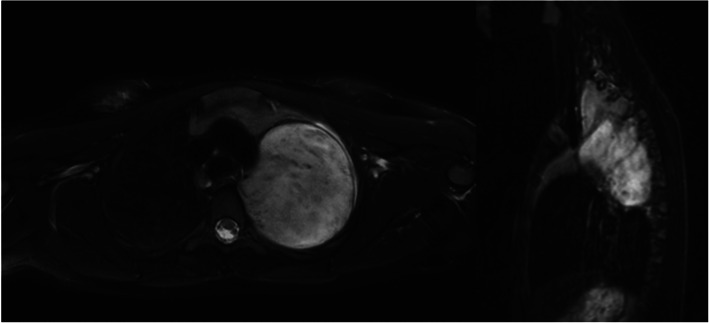
Example of a ganglioneuroma. Figure 1 shows an example of a ganglioneuroma. Axial measurement in T2w imaging resulted in the first reading session in 7.7 cm (reader 1) and 8.1 cm (reader 2), respectively, whereas the maximum three-dimensional diameter was 11.3 cm (reader 1) and 12.3 cm (reader 2). The second reading session resulted in axial diameters of 8.2 cm (reader 1) and 7.6 cm (reader 2) as well as in three-dimensional diameters of 11.0 cm (reader 1) and 11.9 cm (reader 2). Maximum histopathological diameter was 12.3 cm

**Fig. 2:**
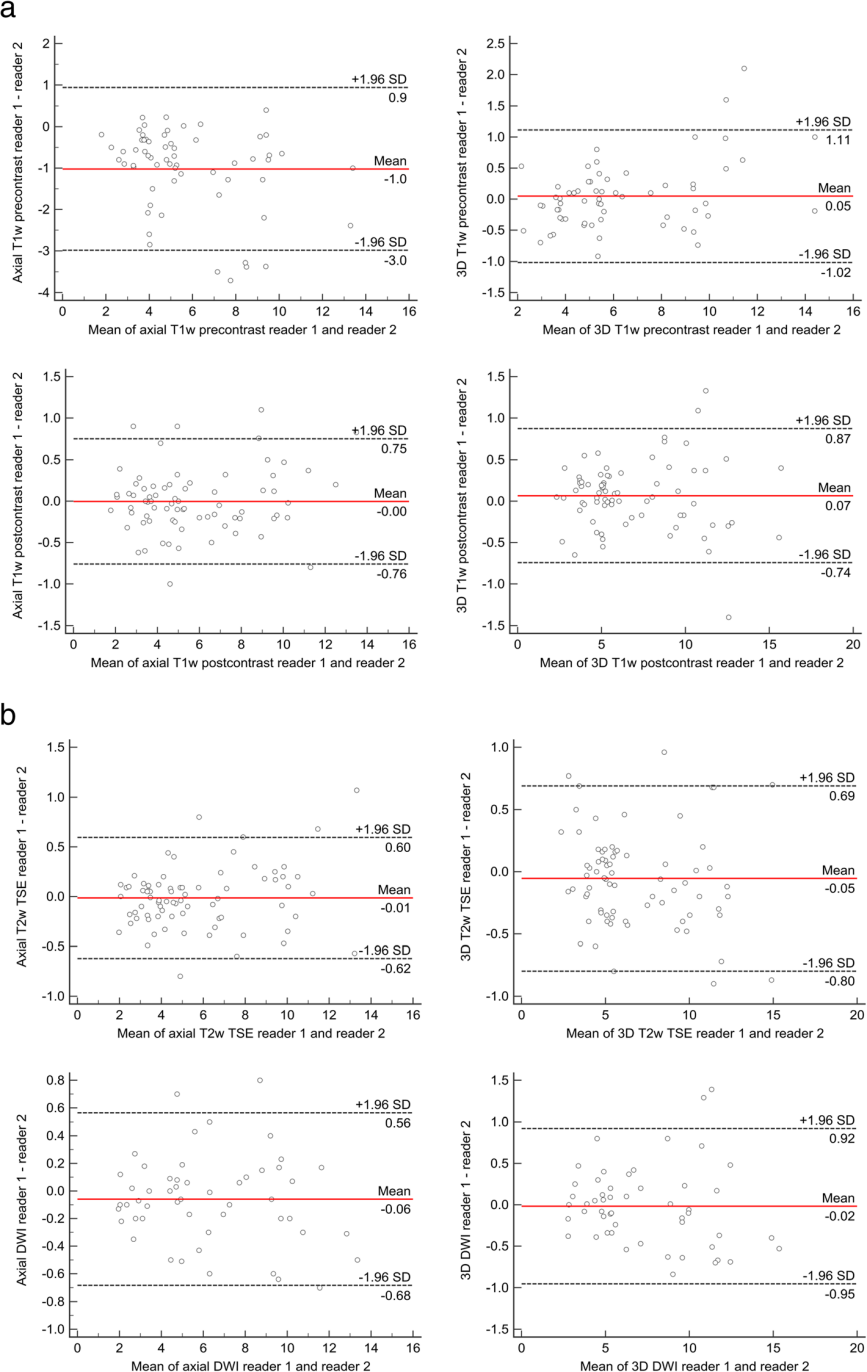
**a** and **b**: Bland-Altman analysis for inter-reader agreement. **a**-**b** show Bland-Altman plots for the comparison of MRI measurements for reader 1 and 2

ICC for inter-reader variability ranged from 0.983 (axial T1w precontrast) to 0.995 (axial DWI). ICC for intra-reader variability of reader 1 ranged from 0.985 (axial T1w precontrast) to 0.993 (3D T1w postcontrast). ICC for intra-reader variability of reader 2 ranged from 0.988 (axial T1w precontrast) to 0.996 (3D T1w postcontrast). Figure 1 shows an example of axial and 3D MRI measurement (Fig. 1). Bland-Altman plots for the comparison of reader 1 and 2 are displayed in Fig.2a and b (Fig. 2a and b).

### Comparison of histopathology and MRI diameters

Due to the high inter-reader agreement of both readers, only the results of reader 1 are displayed in the following. Data of both readers is displayed in Tables 2, 3, and 4. Median histopathological tumor size of the resected specimens was 5.8 cm (IQR: 4.2 cm; range 3.2–16.3 cm). The comparison of the maximum axial diameters revealed a significant difference between measurements of the resected specimens and all applied MRI sequences. Median axial T1w precontrast tumor size was 4.7 cm (median difference: − 1.4 cm; *p* < 0.001). Median axial T1w postcontrast tumor size was 4.9 cm (median difference: − 1.0 cm; p < 0.001). Median axial T2w lesion size was 4.7 cm (median difference: − 1.0 cm; p < 0.001). Median axial DWI tumor size resulted in 5.5 cm (median difference: − 1.3 cm; p < 0.001).

**Table 2:** Comparison of histopathology measurements of the resected specimens with MRI measurements using three-dimensional (3D) and axial measurements according to RECIST

Sequence (n = cases)^a^	Median (IQR) histopathological diameter (cm)	Median (IQR) MRI diameter (cm) Reader 1	Median (IQR) difference (cm) Reader 1	Median (IQR) Absolute error (cm) Reader 1	***p***-value Reader 1
		Reader 2	Reader 2	Reader 2	Reader 2
Axial T1w precontrast (*n* = 31)	5.8 (4.0)	4.7 (3.3)	−1.4 (1.8)	1.1 (1.7)	**< 0.001**
		4.5 (3.6)	−0.8 (1.8)	1.1 (1.7)	**< 0.001**
3D T1w precontrast (n = 31)	5.8 (4.0)	5.4 (5.1)	−0.3 (1.3)	0.7 (0.7)	0.262
		5.3 (5.2)	−0.3 (1.3)	0.8 (0.9)	0.159
Axial T1w postcontrast (*n* = 37)	5.8 (5.2)	4.9 (4.3)	−1.0 (1.9)	1.1 (1.5)	**< 0.001**
		5.0 (4.4)	−0.9 (1.3)	1.0 (1.2)	**< 0.001**
3D T1w postcontrast (n = 37)	5.8 (4.2)	5.5 (4.8)	−0.1 (1.2)	0.6 (0.6)	0.739
		5.5 (4.9)	−0.2 (1.4)	0.7 (0.9)	0.722
Axial T2w TSE (n = 38)	5.8 (4.2)	4.7 (4.2)	−1.0 (1.6)	1.3 (1.7)	**< 0.001**
		4.8 (4.2)	−1.0 (1.6)	1.2 (1.6)	**< 0.001**
3D T2w TSE (n = 38)	5.8 (4.2)	5.4 (5.1)	−0.3 (1.1)	0.6 (0.8)	0.379
		5.4 (5.1)	−0.1 (1.1)	0.5 (0.7)	0.722
Axial DWI (*n* = 26)	6.5 (4.9)	5.5 (6.0)	−1.3 (2.2)	1.4 (2.0)	**< 0.001**
		5.5 (6.0)	−1.2 (2.2)	1.4 (2.0)	**< 0.001**
3D DWI (n = 26)	6.5 (4.9)	6.5 (5.4)	−0.2 (1.3)	0.7 (1,2)	0.159
		6.4 (5.3)	−0.2 (1.5)	0.8 (1.1)	0.196

^a^Not all sequences in all patients acquired

*Abbreviations*: *3D* Three-dimensional

**Table 3:** Correlation analysis of histopathology of the resected specimens and MRI

Sequence (n = cases)^a^	Pearson correlation Reader 1	p-value Reader 1
	Reader 2	Reader 2
Axial T1w precontrast (n = 31)	0.899	< 0.001
	0.895	< 0.001
3D T1w precontrast (n = 31)	0.928	< 0.001
	0.921	< 0.001
Axial T1w postcontrast (n = 37)	0.887	< 0.001
	0.900	< 0.001
3D T1w postcontrast (*n* = 37)	0.896	< 0.001
	0.891	< 0.001
Axial T2w TSE (n = 38)	0.893	< 0.001
	0.883	< 0.001
3D T2w TSE (n = 38)	0.906	< 0.001
	0.901	< 0.001
Axial DWI (n = 26)	0.886	< 0.001
	0.888	< 0.001
3D DWI (*n* = 26)	0.884	< 0.001
	0.891	< 0.001

^a^Not all sequences in all patients acquired

*Abbreviations*: *3D* Three-dimensional

**Table 4:** Influence of neoadjuvant chemotherapy on tumor size measurements

Sequence (n = cases)^a^	Median (IQR) histopathological diameter (cm)	Median (IQR) MRI diameter (cm) Reader 1	Median (IQR) difference (cm) Reader 1	Median (IQR) Absolute error (cm) Reader 1	p-value Reader 1
		Reader 2	Reader 2	Reader 2	Reader 2
*Neoadjuvant chemotherapy*					
Axial T1w precontrast (*n* = 15)	5.8 (2.2)	3.8 (3.4)	−1.0 (1.9)	1.0 (1.9)	**0.001**
		3.9 (3.5)	−0.8 (2.3)	1.1 (2.2)	**0.003**
3D T1w precontrast (n = 15)	5.8 (2.2)	5.2 (2.9)	−0.3 (1.8)	0.7 (1.0)	0.224
		5.2 (2.6)	−0.5 (2.1)	0.8 (1.2)	0.271
Axial T1w postcontrast (n = 18)	5.9 (3.9)	4.3 (4.5)	−1.0 (2.1)	1.1 (1.8)	**0.001**
		4.6 (4.4)	−1.0 (1.3)	1.0 (1.2)	**< 0.001**
3D T1w postcontrast (n = 18)	5.9 (3.9)	5.3 (3.9)	−0.1 (1.3)	0.7 (0.8)	0.369
		5.2 (4.2)	−0,3 (1.7)	0.7 (1.1)	0.387
Axial T2w TSE (n = 19)	5.8 (3.8)	4.1 (3.6)	−1.5 (1.9)	1.5 (1.8)	**< 0.001**
		4.2 (3.6)	−1.3 (1.8)	1.4 (1.7)	**0.001**
3D T2w TSE (*n* = 19)	5.8 (3.8)	5.1 (3.4)	−0.4 (1.7)	0.6 (1.2)	0.101
		5.2 (3.5)	−0.1 (1.3)	0.6 (1.3)	0.341
Axial DWI (*n* = 14)	6.2 (4.0)	4.8 (6.4)	−1.5 (2.1)	1.5 (2.0)	**0.001**
		4.6 (5.8)	−1.4 (2.1)	1.4 (2.0)	**0.001**
3D DWI (n = 14)	6.2 (4.0)	5.2 (5.0)	−0.5 (1.7)	0.7 (1.2)	0.100
		5.4 (5.0)	−0.6 (1.6)	0.9 (1.1)	0.094
*No neoadjuvant chemotherapy*					
Axial T1w precontrast (n = 16)	5.5 (4.9)	5.0 (3.5)	−1.1 (1.8)	1.1 (1.7)	**< 0.001**
		5.3 (4.1)	−1.0 (1.4)	1.0 (1.4)	**< 0.001**
3D T1w precontrast (n = 16)	5.5 (4.9)	5.7 (5.2)	−0.3 (1.4)	0.6 (0.5)	0.792
		5.7 (5.3)	−0.2 (1.5)	0.7 (0.6)	0.308
Axial T1w postcontrast (n = 19)	5.8 (5.2)	5.3 (4.1)	−0.9 (1.8)	1.1 (1.2)	**0.002**
		5.3 (4.3)	−0.9 (1.7)	1.0 (1.3)	**0.001**
3D T1w postcontrast (n = 19)	5.8 (5.2)	6.2 (5.7)	0.1 (1.5)	0.6 (0.6)	0.658
		6.0 (5.5)	−0.1 (1.3)	0.7 (0.9)	0.914
Axial T2w TSE (n = 19)	5.8 (5.2)	5.0 (4.1)	−0.8 (1.5)	1.0 (1.5)	**0.001**
		5.2 (3.9)	−0.9 (1.6)	1.0 (1.6)	**0.003**
3D T2w TSE (n = 19)	5.8 (5.2)	6.2 (5.7)	0.2 (1.5)	0.7 (0.8)	0.686
		6.2 (5.7)	−0.1 (1.3)	0.5 (0.5)	0.521
Axial DWI (*n* = 12)	8.1 (6.4)	6.6 (4.7)	−1.1 (2.7)	1.4 (2.4)	**0.043**
		6.8 (4.8)	−1.0 (2.6)	1.4 (2.1)	0.062
3D DWI (*n* = 12)	8.1 (6.4)	9.0 (6.2)	−0.2 (1.6)	0.7 (1.2)	0.745
		9.5 (6.3)	0.0 (1.4)	0.6 (1.0)	0.984

^a^Not all sequences in all patients acquired

*Abbreviations*: *3D* Three-dimensional

There was no significant difference between 3D MRI based lesion size measurements and the resected specimens regardless of the applied sequence. Median 3D T1w precontrast tumor size was 5.4 cm with a median difference of − 0.3 cm (*p* = 0.262). Median 3D T1w postcontrast tumor size was 5.5 cm (median difference: − 0.1 cm; *p* = 0.739). Median 3D T2w tumor size resulted in 5.4 cm (median difference: − 0.3 cm; *p* = 0.379). Median 3D DWI based tumor size was 6.5 cm (median difference: − 0.2 cm; *p* = 0.159). Further details for reader 1 and 2 are displayed in Table 2.

There was almost perfect Pearson correlation (> 0.85) between histopathology measurements of the resected specimens and MRI measurements regardless of the sequence (Table 3).

### Bland-Altman assessment

**Fig. 3:**
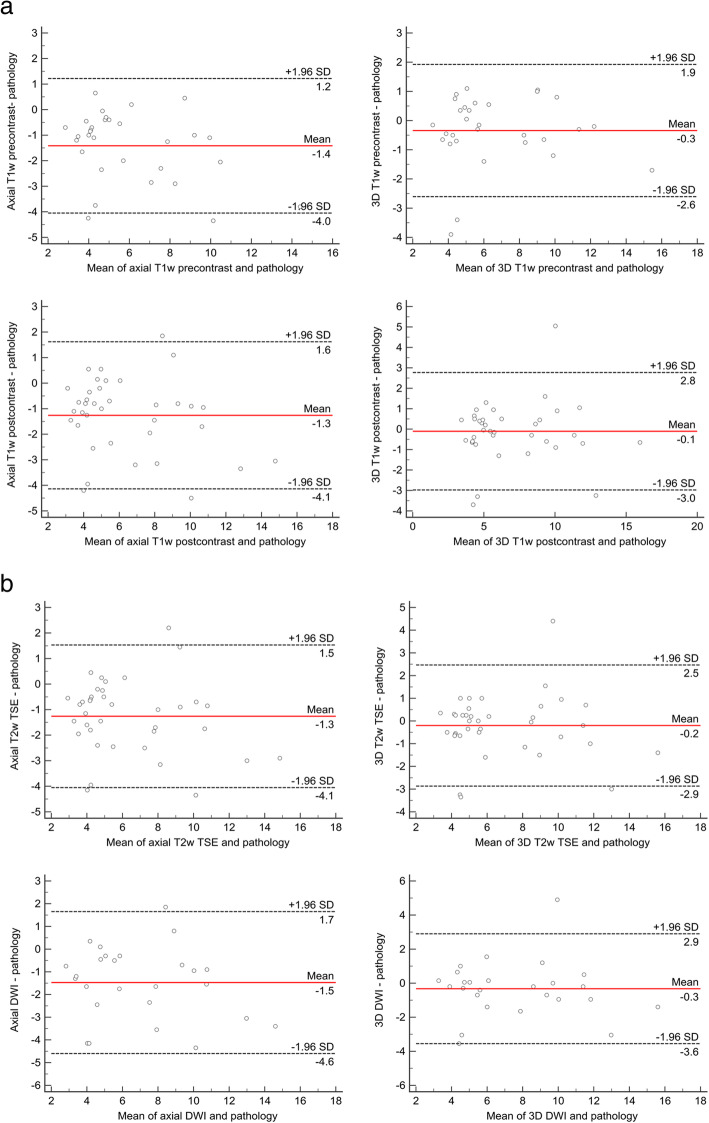
**a** and **b**: Bland-Altman analysis for agreement of histopathology and MRI measurements. **a**-**b** show Bland-Altman plots for the comparison of histopathology and MRI (reader 1) measurements

The Bland-Altman assessment provided a systematic underestimation for all sequences and for both readers. Lowest mean difference was found for reader 1 in 3D T1w postcontrast (− 0.1 cm) and for reader 2 in 3D T2w TSE (− 0.1 cm). Bland-Altman plots for reader 1 are displayed in Fig. 3a and b (Fig. 3a and b).

### Influence of neoadjuvant therapy

Eighteen patients with 19 lesions (NB: *n* = 11; GNB: *n* = 8) received neoadjuvant chemotherapy prior to surgery. Nineteen patients (NB: *n* = 6; GNB: *n* = 3; GN: *n* = 10) underwent gross total resection without preoperative chemotherapy. Median tumor size after neoadjuvant chemotherapy according to the histopathology report was 5.8 cm (IQR: 3.8 cm) vs. 5.8 cm (IQR: 5.2 cm) without chemotherapy (*p* = 0.817).

In the group of patients with neoadjuvant chemotherapy, 3D T1w precontrast measurements resulted in a median of 5.2 cm with a median difference of − 0.3 cm (*p* = 0.224). Three-dimensional T1w postcontrast measurements achieved slightly higher results with a median of 5.3 cm (median difference: − 0.1 cm; *p* = 0.369). Three-dimensional T2w measurements had similar results with a median of 5.1 cm (median difference: − 0.4 cm; *p* = 0.101). There was also no significant difference in 3D DWI tumor margins with a median diameter of 5.2 cm (median difference: − 0.5 cm; *p* = 0.100). Similar to the overall assessment, all axial measurements were significantly different from the resected specimens (Table 4).

In patients without neoadjuvant therapy the comparison of lesion size between the resected specimens and MRI measurements showed no significant difference in 3D measurements whereas all axial measurements (except axial DWI for reader 2) were significantly different. Further details are displayed in Table 4.

## Discussion

This study demonstrated that there is a significant difference between all axial MRI measurements and measurements of the resected specimens in neuroblastic tumors independently of the applied sequence. However, by using the maximum 3D diameter no significant difference could be found, regardless of the applied MRI sequence. Additionally, there was almost perfect correlation between all MRI and resected specimens’ measurements independently of the measurement approach.

Our results indicate a systematic underestimation of the tumor size in all applied MRI sequences. Previously published reasons for this systematic underestimation might be calcifications as well as fibrosis within the tumor tissue [16]. However, we think that due the lowest mean difference in the Bland-Altman assessment, T2w TSE or T1w postcontrast imaging should be used for lesion size measurements. DWI provided in our study after Bland-Altman assessment larger differences, however without significant difference compared to the resected specimens using the 3D approach. This might be due to the inhomogeneity of neuroblastic tumors and edema blurring the exact tumor margins. Additionally, chemotherapy has to be taken into account for the difference between MRI and histopathology measurements of the resected specimens although in both treatment groups no significant difference between 3D measurements and histopathology could be found. Our results are in line with the data reported by Trout et al. who demonstrated that one-dimensional and also 3D measurements underrepresent tumor response in comparison to volumetry [17]. However, it was also previously shown that the different measurement approaches do not affect the outcome of the patients [15, 18].

In this context, the question of the importance of exact measurements arises, of course. Exact tumor size determination before operation is vital for the assessment of residual tumor in postoperative imaging studies. Large discrepancy between the MRI report and the histopathological report might cause confusion postoperatively, especially if the resected specimen is smaller than it was stated in the radiological report. This situation could alert the responsible surgeon and arise the suspicion of incomplete resection. Furthermore, for postoperative imaging control the correctness of measurements plays also a key role for follow-up and relapse evaluation. Ideally, volumetric approaches should be followed as it was previously demonstrated to be the most accurate analysis tool [17]. However, as volumetry is a very time-consuming task mostly only the largest diameter is used for comparison. Therefore, the correctness of this parameter is of utmost importance. The significant difference of the axial RECIST measurements compared to the resected specimens are likely due to tumor shape. Most neuroblastic tumors are not characterized by a perfectly round shape but are larger in one axis. Therefore, the assessment of the largest diameter displays the smallest margin of error.

Due to the low incidence of neuroblastic tumors, our sample size is relatively small. However, for a monocentric study it still represents one of the largest study cohorts and to our knowledge the first one with comparison of lesion size with histopathology as standard of reference. As our hospital displays a national reference center for neuroblastic tumors many patients were only referred to surgery with the lack of a uniform imaging protocol. Additionally, this led in some cases to an unavoidable delay between the most current MRI examination and surgery as due to the risks involved, no MRI examination was repeated in our center if not absolutely necessary for surgery. Further, ideally multicentric prospective studies with a uniform imaging protocol are necessary to evaluate these initial results.

## Conclusions

The results of this study indicate that there is a strong correlation between MRI and histopathology measurements of the resected specimens. The lowest mean difference between MRI and histopathology was found in three-dimensional measurements in T2w TSE and T1w postcontrast images. Therefore, these both sequences might be most suitable for lesion size determination of neuroblastic tumors.

## Data Availability

The datasets generated and/or analysed during the current study are not publicly available due to privacy and ethical restriction but are available from the corresponding author on reasonable request.

## Abbreviations

NB: Neuroblastoma
GNB: Ganglioneuroblastoma
GN: Ganglioneuroma
INRG: International Neuroblastoma Risk Group
INRGSS: International Neuroblastoma Risk Group classification system and staging system
US: Ultrasound
CT: Computed tomography
MRI: Magnetic resonance imaging
3D: Three-dimensional
RECIST: Response Evaluation Criteria In Solid Tumors
DWI: Diffusion-weighted imaging
TSE: Turbo spin echo
